# Determinants of ethnic differences in the uptake of child healthcare services in New Zealand: a decomposition analysis

**DOI:** 10.1186/s12939-022-01812-3

**Published:** 2023-01-16

**Authors:** Sonia Lewycka, Kabir Dasgupta, Alexander Plum, Terryann Clark, Mary Hedges, Gail Pacheco

**Affiliations:** 1 Centre for Tropical Medicine and Global Health, Nuffield Department of Medicine, University of Oxford, Oxford, UK; 2 Oxford University Clinical Research Unit, National Hospital for Tropical Diseases, 78 Giai Phong, Dong Da District, Hanoi, Vietnam; 3 Faculty of Business, Economics and Law, NZ Work Research Institute, Auckland University of Technology, Auckland, New Zealand; 4 School of Nursing, Faculty of Medical and Health Sciences, University of Auckland, Auckland, New Zealand; 5 Mahitahi Hauora Primary Health Entity, Whangārei, New Zealand

**Keywords:** Ethnic health disparities, Structural racism, Primary care, Healthcare uptake, Determinants, Indigenous, Māori, Pacific, Asian, New Zealand

## Abstract

**Background:**

There are persistent ethnic gaps in uptake of child healthcare services in New Zealand (NZ), despite increasing policy to promote equitable access. We examined ethnic differences in the uptake of immunisation and primary healthcare services at different ages and quantified the contribution of relevant explanatory factors, in order to identify potential points of intervention.

**Methods:**

We used data from the Growing Up in New Zealand birth cohort study, including children born between 2009 and 2010. Econometric approaches were used to explore underlying mechanisms behind ethnic differences in service uptake. Multivariable regression was used to adjust for mother, child, household, socioeconomic, mobility, and social factors. Decomposition analysis was used to assess the proportion of each ethnic gap that could be explained, as well as the main drivers behind the explained component. These analyses were repeated for four data time-points.

**Results:**

Six thousand eight hundred twenty-two mothers were enrolled during the antenatal survey, and children were followed up at 9-months, 2-years and 4-years. In univariable models, there were ethnic gaps in uptake of immunisation and primary care services. After adjusting for covariates in multivariable models, compared to NZ Europeans, Asian and Pacific children had higher timeliness and completeness of immunisation at all time-points, while indigenous Māori had lower timeliness of first-year vaccines despite high intentions to immunise. Asian and Pacific mothers were less likely to have their first-choice lead maternity caregiver (LMC) than NZ Europeans mothers, and Māori and Asian mothers were less likely to be satisfied with their general practitioner (GP) at 2-years. Healthcare utilisation was strongly influenced by socio-economic, mobility and social factors including ethnic discrimination. In decomposition models comparing Māori to NZ Europeans, the strongest drivers for timely first-year immunisations and GP satisfaction (2-years) were household composition and household income. Gaps between Pacific and NZ Europeans in timely first-year immunisations and choice of maternity carer were largely unexplained by factors included in the models.

**Conclusions:**

Ethnic gaps in uptake of child healthcare services vary by ethnicity, service, and time-point, and are driven by different factors. Addressing healthcare disparities will require interventions tailored to specific ethnic groups, as well as addressing underlying social determinants and structural racism. Gaps that remain unexplained by our models require further investigation.

**Supplementary Information:**

The online version contains supplementary material available at 10.1186/s12939-022-01812-3.

## Background

Interactions across various social determinants of health, including economic conditions, education, cultural identity, demographic attributes, and access to public services, cumulatively determine the burden of disease and health disparities across and within countries [1]. As such, a population’s overall wellbeing not only depends on the quality of public healthcare services rendered, but also relies on investments in social services that can reduce socio-economic disparities within a society [2].

Current policy in New Zealand (NZ) aims to achieve equitable access for healthcare services, by ensuring that cost is not a barrier to uptake [3]. Antenatal care and health services for children aged under 14-years, including the national vaccination schedules, are free of charge to all residents. However, structural racism is embedded in long-standing social policy [4], and despite these initiatives, large social and ethnic disparities in healthcare coverage persist particularly for Māori. Colonisation has had a significant and ongoing impact for Māori, the Indigenous peoples of NZ, despite rights and obligations guaranteed in Te Tiriti o Waitangi (Treaty of Waitangi 1840). Ethnic and Indigenous differences in usage of preventive healthcare services particularly exposes Māori and other ethnic minority populations to greater risk of future health disorders.

The 2018–19 New Zealand Health Survey revealed that Māori and Pacific children had a higher unmet need for primary healthcare [5]. Important underlying reasons for such gaps included lack of transport, cost, and low levels of trust and confidence in the child’s GP. A similar pattern is found internationally, with lower utilisation of healthcare services among ethnic minority groups [6–8], and particularly for indigenous ethnic groups [9]. In the US, children from racial minority and marginalised communities were found to have poorer health status and disrupted access to care [10].

Underutilisation of preventive healthcare services during early childhood may lead to future risks of adverse health outcomes [11–14]. Relative to other ethnic groups, Māori and Pacific pre-school children have higher rates of hospitalisation resulting from infectious diseases. For Pacific children, this was strongly associated with delayed immunisation [15]. Disproportionate rates of hospitalisation could potentially be avoided with earlier primary care visits [16]. Māori and Asian mothers also have worse maternal and perinatal outcomes than NZ European/European mothers, and lack of engagement with maternity care was one of the main determinants identified [17]. A systematic review also found that ethnic differences in pre-term birth can be explained by gaps in quality, continuity, and trust in maternity care providers [18].

Apart from ethnic disparities, healthcare usage is also considerably lower for economically disadvantaged groups [5]. This suggests that the observed ethnic gaps may be driven by differences in ethnicity-specific socio-economic characteristics. An analysis of ethnic disparities in health and wellbeing for Māori adolescents showed that many ethnic gaps were substantially reduced after adjusting for individuals’ socio-economic status [19].

Social and economic factors that are found to be associated with child healthcare uptake include parental education and economic wellbeing [6, 7, 20, 21]; family structure [21–23]; immigration status [6, 8, 21, 24]; locational characteristics and residential mobility [25, 26]; and societal factors such as social stigma and demographic biases [27–29]. Importantly, there are also ethnic inequalities in many of these social determinants of health.

Although previous studies have explored descriptive evidence on ethnic disparities in the uptake of healthcare services, only a few go further and attempt to explain the differences by accounting for various individual-level characteristics that may be of relevance. This analysis aims to examine ethnic differences in uptake of immunisation and primary care, and to quantify the contribution of relevant social determinants to explaining these differences.

## Methods

### Study population and data sources

We obtained data from the Growing Up in New Zealand (GUiNZ) birth cohort study, which includes children born between 2009 and 2010. This study is co-ordinated by the University of Auckland’s Centre for Longitudinal Research – He Ara ki Mua, and the study cohort has been described in detail elsewhere [30]. In brief, a total of 6,822 mothers were enrolled during the antenatal survey, which was conducted across Auckland, Northland, and Waikato regions in New Zealand. The birth characteristics of the cohort are aligned well with all NZ births over the period 2007 to 2010 [31]. The GUINZ data uses a series of computer-assisted face-to-face and telephone interviews. We used four data collection waves (DCW) for interviews carried out before childbirth (DCW0; antenatal); nine-months post-childbirth (DCW1); when the child was 2-years old (DCW2); and 4-years old (DCW4).

### Variables

For each wave, we derived survey-specific variables related to two broad outcomes of interest. Table 1 shows definitions and descriptive statistics of these outcome variables, along with information on their respective waves. All outcomes were binary. We used four main outcome indicators related to immunisation: antenatal intention to immunise; timeliness of registered immunisation by 9-months; self-reported immunisation by 15-months; and self-reported immunisation by 4-years. The 9-month survey provides both self-reported child immunisation information as well as a more objective measurement from administrative vaccination records from the National Immunisation Register (NIR). We used four variables related to primary care: whether the type of Lead Maternity Carer (LMC) was the first choice; if the mother had seen a family doctor or GP since becoming pregnant; satisfaction with child’s usual GP at 9-months; and satisfaction with usual GP at 2-years. In DCW0 (the antenatal data collection wave), survey respondents were first asked what type of LMC they have. The categories available were GP, independent midwife, hospital midwife, obstetrician, shared care GP and midwife, and other. After this question, respondents were then asked whether the type they had was their first choice. Note that in NZ, GP and LMC are often separate, so if mothers are otherwise well throughout pregnancy, they are unlikely to need to see a GP. LMC visits are free, while regular GP visits are not.

**Table 1:** Definitions of outcome measures, ethnicity variables and covariates

	Variable	Data collection wave	Definition	Proportion/mean	N
Outcome measures					
Immunisation	Antenatal intention to fully immunise	DCW0	Dummy variable: 1 if intended to have child fully immunised; 0 otherwise	0.81	6171
	Self-reported immunisation	DCW1	Dummy variable: 1 if mother reported that the 6-week, 3-month and 5-month immunisations were all given; 0 otherwise	0.84	6846
	All first-year immunisations	DCW1	NIR-validated dummy variable: 1 if the 6-week, 3-month and 5-month immunisations were all given; 0 otherwise	0.88	6668
	All first-year immunisations on time^1^	DCW1	NIR-validated dummy variable: 1 if the 6-week, 3-month and 5-month immunisations were all given on time; 0 otherwise	0.70	6668
	Received 15-month immunisations	DCW2	Dummy variable: 1 if mothers reported that child received their 15-month immunisation (partial or full); 0 otherwise	0.94	6312
	Received age 4 immunisations	DCW4	Dummy variable: 1 if mother reported that child received their age 4 immunisations (partial or full); 0 otherwise	0.87	5951
Primary care	Choice of LMC^2^ (antenatal)	DCW0	Dummy variable: 1 if type of LMC was first choice; 0 otherwise	0.89	6645
	Seen GP or any health professionals since becoming pregnant (antenatal)	DCW0	Dummy variable: 1 if the mother has seen any family doctor or GP since she became pregnant; 0 otherwise	0.72	6821
	Satisfied with usual GP practice (age 9-months)	DCW1	Dummy variable: 1 if very or completely satisfied with child's usual GP practice; 0 otherwise	0.68	6252
	Satisfied with usual GP practice (age 2)	DCW2	Dummy variable: 1 if very or completely satisfied with child's usual GP practice; 0 otherwise	0.67	5998
Ethnicity and covariates					
Ethnicity	NZ European	DCW0	Binary indicator for NZ European in self-prioritised ethnicity	0.550	6464
	Māori	DCW0	Binary indicator for Māori in self-prioritised ethnicity	0.133	6464
	Pacific peoples	DCW0	Binary indicator for Pacific peoples in self-prioritised ethnicity	0.136	6464
	Asian	DCW0	Binary indicator for Asian in self-prioritised ethnicity	0.144	6464
	Other ethnicities	DCW0	Binary indicator for other ethnicities not defined above	0.037	6464
Mother	First child	DCW0	Binary indicator for whether child is the first born	0.420	6464
	Current age (years)	DCW0 – 4	Continuous measure of mother’s current age (in years)	30.2	6464
	Current weight (kg)	DCW0	Continuous measure of mother’s current weight (in kg)	80.9	5316
	Disability status	DCW0 – 4	Binary indicator for whether mother has a long-term physical disability	0.057	6464
	Smokes regularly	DCW0 – 4	Binary indicator for whether mother smokes regularly	0.101	5840
Child	Girl	DCW1	Binary indicator for whether child is a girl	0.484	6464
	Birthweight (grams)	DCW1	Continuous measure of child’s birthweight (in grams)	3484.9	6460
	Child health/developmental problem	DCW1—4	Binary indicator for whether child has a health/developmental problem	0.094	6458
	Child health concern	DCW4	Binary indicator for whether there is general health concern for the child (e.g. vision, speech, behaviour, mobility etc.)	0.365	5802
	Common child illness	DCW4	Binary indicator for whether child has had at least one common illness (e.g. wheezing, coughing, ear infections etc.)	0.740	5801
Socio-economic	Mother’s employment	DCW0 – 4	Binary indicator of mother’s employment (equals 1 if employed in a job for wages and salaries; 0 otherwise)	0.551	6464
	Mother’s post-graduate qualification	DCW0	Binary indicator for whether mother has a post-graduate qualification (e.g. Honours, Masters, Doctorate)	0.161	6446
	Household income < NZ$ 50 K	DCW0 – 4	Binary indicator for household income less than NZ$ 50,000	0.225	4979
	Household income > = NZ$ 50 K & < = 100 K	DCW0 – 4	Binary indicator for household income between NZ$ 50,000 and NZ$ 100,000	0.401	4979
Household	Partner	DCW0 – 4	Binary indicator for whether mother currently has a partner (equals 1 if mother currently has a partner; 0 otherwise)	0.856	6464
	NZ born mother	DCW0	Binary indicator derived from mother’s country of birth (equals 1 if NZ; 0 otherwise)	0.648	6464
	Household size	DCW0	Continuous measure of number of people residing in the same household as mothers	2.757	6441
	Number of people > = 18 years	DCW1	Continuous measure of number of people aged 18 years or over residing in the same household as mother	1.523	6464
	Number of people < 18 years	DCW1	Continuous measure of number of people aged under 18 years residing in the same household as mother	2.123	6461
	Number of siblings	DCW2 – 4	Continuous measure of number of siblings	1.129	6406
Mobility	Mother self-driving	DCW0 – 4	Binary indicator for mother’s main personal transport (equals 1 if drove private/company car; 0 otherwise)	0.784	5838
	Numbers of residential moves	DCW0 – 4	Continuous measure of number of times mother moved in the past 5 years	2.319	6443
	Local healthcare	DCW1	Binary indicator for whether most of household’s healthcare visits are in local areas	0.744	6454
	Rural location	DCW0 – 4	Binary indicator for whether mother lives in a rural area	0.071	6464
	Internet access	DCW4	Binary indicator for whether household has internet access from home	0.918	5804
Social	Discriminated against	DCW0 – 4	Binary indicator if mother was a victim in the past of ethnically motivated unfair treatment (physical, verbal, and/or by a health professional)	0.220	6464
	Discouraged to immunise	DCW0	Binary indicator if during pregnancy mother received any information that discouraged her to immunise child	0.128	6464
	Encouraged to immunise	DCW0	Binary indicator if during pregnancy mother received any information that encouraged her to immunise child	0.352	6464
	Childcare services	DCW1 – 4	Binary indicator if child goes to any childcare services	0.133	6464

We use four data collection waves (DCW) from GUINZ that represent interviews that were carried out before childbirth (DCW0; antenatal); nine months post-childbirth (DCW1); when the child was 2 years old (DCW2); and 4 years old (DCW4). Where a variable is used in multiple data collection waves, the sample size provided is for the first available wave

1: Grant et al. (2016) define immunisation on time as vaccination received within 30 days of their due date; 2: LMC = Lead Maternity Carer

Table 1 also shows the definitions of covariates. Ethnicity was defined based on self-prioritised ethnicity using four main categories; NZ European, Māori, Pacific peoples, and Asian, as well as other ethnicities that included Middle Eastern, Latin American, and African (MELAA), and other ethnic groups. The survey-specific covariates used in this study were grouped into: mother and child characteristics (incorporating information on child and mother’s health condition), socio-economic status (including mothers’ education, employment and household income level); household attributes (including household size, whether mother was born in NZ, and partnership status); mobility (including location, residential moves, and access to personal transport); and social aspects (including experience of discrimination, discouragement or encouragement regarding vaccination, and childcare).

Covariates from other time points (DCW1, DCW2 and DCW4) were added where a particular survey wave provided further information deemed useful to include as a predictor of healthcare service uptake. The most recent available update was used for each variable, except for ethnicity and mother’s education, which were time invariant. All survey waves post-birth (DCW1, DCW2, and DCW4) include more details about the child, such as their sex, and whether they had ongoing health concerns. DCW1 also permits the split of one continuous indicator that captures household size in DCW0 into two continuous indicators for number of people aged under 18 in the household, and number of people aged 18 or over in the household. Further to that, DCW2 and DCW4 allow inclusion of number of child’s siblings in the household.

### Statistical analysis

A detailed description of the statistical method is available in the Supplementary material. First, for binary outcomes we used a non-linear (probit) regression to explore the relationships between each of the outcomes and the sets of covariates and identify ethnic differences in the uptake of healthcare. We present these as marginal effects. Next, we quantified the contribution of each of these sets of factors to understand what proportion of the ethnic gap they explained. We evaluated the contribution of the different covariates in explaining observed ethnic differences in indicators of health service uptake using the Fairlie decomposition method which extends the standard Blinder-Oaxaca decomposition method for application in non-linear models [32].

## Results

### Participant characteristics

The total numbers of participants in each data collection wave were: 6,822 for the antenatal survey (DCW0), 6,389 for the 9-month survey (DCW1), 6,508 for the 2-year survey (DCW2), and 5,899 for the 4-year survey (DCW3). Descriptive results are presented in Table 1. In the antenatal survey, the proportion of NZ European participants was 0.55, Māori 0.13, Pacific 0.14 Asian 0.14 and other ethnicities 0.04. The number of participants included in each analysis differed, depending on wave-specific participation coverage and data completeness for covariates. Participants with missing data were excluded from regression analyses.

### Determinants of healthcare uptake

#### Immunisation

At the antenatal survey, 81% of 4520 mothers in our regression sample intended to immunise their children. Unadjusted analyses showed higher levels of intention to immunise among all ethnic groups compared to NZ Europeans (Table 2). Looking at actual immunisation, 71% of 5384 children received their first-year immunisations on time, with Māori and Pacific children having lower proportions and Asian children having a higher proportion than NZ Europeans. However, Pacific children appear to be marginally more likely to be immunised on time than NZ European children when regressions are adjusted for individual characteristics. At 2-years, the proportion of Asian and Pacific children fully immunised was higher than NZ Europeans, and the gaps between ethnic groups were smaller. By the 4-year survey, the complete immunisation rate was 86% (of 4844 mothers). In the unadjusted model, higher immunisation coverage for Asian and Pacific children persisted, while Māori children had lower likelihood of being immunised. However, the difference in 4-year immunisation rates between Māori and NZ European children is statistically insignificant in the adjusted model.

**Table 2:** Probit regression results for immunisation variables

		Antenatal intention to fully immunise				All first-year immunisations on time				Received 15-month immunisations				Received age 4 immunisations			
		Unadjusted model		Adjusted model		Unadjusted model		Adjusted model		Unadjusted model		Adjusted model		Unadjusted model		Adjusted model	
		SM = 0.809				SM = 0.708				SM = 0.984				SM = 0.864			
Ethnicity	Māori	0.053^c^	(0.018)	0.008	(0.019)	-0.151^c^	(0.018)	-0.061^c^	(0.019)	0.002	(0.005)	0.000	(0.006)	-0.034^b^	(0.014)	-0.009	(0.016)
	Pacific peoples	0.239^c^	(0.024)	0.124^c^	(0.028)	-0.052^c^	(0.019)	0.039^a^	(0.023)	0.037^c^	(0.013)	0.034^c^	(0.012)	0.061^c^	(0.019)	0.076^c^	(0.021)
	Asian	0.168^c^	(0.019)	0.140^c^	(0.023)	0.153^c^	(0.020)	0.088^c^	(0.025)	0.024^c^	(0.008)	0.022^b^	(0.010)	0.124^c^	(0.019)	0.082^c^	(0.022)
	Other ethnicity	0.082^c^	(0.032)	0.065^b^	(0.032)	0.068^a^	(0.036)	0.066^a^	(0.035)	-0.001	(0.009)	-0.001	(0.009)	0.034	(0.029)	0.032	(0.029)
Mother	Current age (years)			-0.005^c^	(0.001)			0.002^b^	(0.001)			0.000	(0.000)			0.000	(0.001)
	Current weight (kg)			0.001^b^	(0.000)			-	-			-	-				
	Disability status			-0.004	(0.023)			0.025	(0.029)			-0.002	(0.008)			-0.003	(0.022)
	Smokes regularly			0.046^a^	(0.024)			-0.026	(0.019)			0.002	(0.006)			0.003	(0.017)
Child	Girl			-	-			0.013	(0.012)			0.002	(0.003)			0.001	(0.010)
	Birthweight (grams)			-	-			0.00002^a^	(0.000)			-0.00001^a^	(0.000)			0.00002^b^	(0.000)
	First child			-	-			0.088^c^	(0.015)			0.011^b^	(0.005)			0.055^c^	(0.011)
	Child health/developmental concern			-	-			-0.049^c^	(0.019)			-0.002	(0.006)			0.000	(0.010)
	Common child illness			-	-			-	-							0.005	(0.011)
Socio-economic	Mother’s employment			-0.002	(0.013)			-0.016	(0.013)			0.000	(0.004)			0.016^a^	(0.010)
	Mother’s post-graduate qualification			0.022	(0.015)			0.001	(0.017)			0.000	(0.005)			0.017	(0.014)
	Household income < NZ$ 50 K (*Omitted* > *100 K*)			-0.033^a^	(0.019)			-0.099^c^	(0.018)			-0.007	(0.006)			-0.03	(0.023)
	Household income > = NZ$ 50 K & < = 100 K			-0.024^a^	(0.013)			-0.070^c^	(0.015)			0.000	(0.004)			-0.031	(0.024)
Household	Partner			-0.013	(0.033)			0.013	(0.024)			-0.009	(0.008)			0.030^a^	(0.017)
	NZ born mother			-0.039^c^	(0.015)			-0.030^a^	(0.016)			0.001	(0.005)			-0.017	(0.014)
	Household size (number)			0.007	(0.005)				-			-	-				
	Number of people > = 18 years			-	-			0.000	(0.007)			-	-				
	Number of people < 18 years			-	-			-0.069^c^	(0.006)			-	-				
	Number of siblings			-	-			-	-			0.003	(0.002)			-0.014^c^	(0.004)
Mobility	Numbers of residential moves			-0.012	(0.003)			-0.012^c^	(0.004)			-0.001	(0.003)			-0.013^c^	(0.005)
	Rural location			-0.048^c^	(0.019)			-0.019	(0.021)			-0.009^a^	(0.005)			-0.031^b^	(0.015)
	Local healthcare			-	-			0.036^c^	(0.013)								
	Mother self-driving			0.034^b^	(0.016)			-0.005	(0.018)			-0.021^b^	(0.010)			0.024	(0.016)
	Internet access			-	-			-	-							-0.031	(0.021)
Social	Discriminated against			-0.02	(0.013)			-0.011	(0.014)			-0.002	(0.006)			-0.02	(0.018)
	Discouraged to immunise			-0.144^c^	(0.014)			-0.147^c^	(0.017)			-0.018^c^	(0.005)			-0.065^c^	(0.014)
	Encouraged to immunise			0.052^c^	(0.012)			0.015	(0.013)			0.009^b^	(0.004)			0.006	(0.011)
	Childcare services			-	-			0.045^c^	(0.018)			0.008^b^	(0.004)			0.069^c^	(0.019)
	Observations	4520		4520		5384		5384		5143		5143		4844		4844	

The above table presents marginal effects from Probit models. The robust standard errors are reported within parentheses

*SM* Sample mean

^c^, ^b^, ^a^denote the coefficients are significantly different from zero at the 1%, 5%, and 10% level respectively. The ethnicity information is derived from self-prioritised ethnicity

Overall Pacific and Asian children had higher immunisation intention and uptake across all four waves. These findings are supported by negative marginal effects in the antenatal and 9-month waves for ‘NZ born’ mothers who are mostly NZ European and Māori. 

Other factors associated with lower immunisation uptake were having lower household income, larger household size, and being discouraged to immunise. Factors associated with higher uptake were being the first-born child, attending childcare services, and being encouraged to immunise.

We also conducted ethnic and age-specific regressions for the timeliness of first-year immunisations (Supplementary Tables A.1 and A.2). The positive effect associated with the child being first born was consistent across ethnic groups, while discouragement to immunise played a stronger role for NZ European and Māori, than for Pacific and Asian groups.

When comparing regression analyses for administrative immunisation records (NIR) and self-reported child immunisation (Supplementary Table A.3) the results were qualitatively similar, which suggests that our analysis was not affected by reporting biases.

#### Primary care uptake and satisfaction

At the antenatal survey, 79% of 4527 mothers had seen a GP while pregnant. Both unadjusted and covariate-adjusted analyses show that this was higher for Pacific and Asian mothers than NZ Europeans (Table 3). The likelihood of being able to consult the first-choice lead maternity carer (LMC) was lower for Pacific and Asian mothers. At 9-months, 68% of 5341 mothers were satisfied with their child’s GP, and this was higher for Pacific mothers but lower for Māori mothers, when compared to NZ Europeans. However, there was no statistical difference between Māori and NZ European mothers in the multivariable model. Finally, at 2-years, the proportion of mothers satisfied with child’s GP was lower for Māori and Asian mothers.

**Table 3:** Probit regression results for primary care variables

		Seen doctor / GP while pregnant				First choice LMC				Satisfied with usual GP practice (age 9 months)				Satisfied with usual GP practice (age 2 years)			
		Unadjusted model		Adjusted model		Unadjusted model		Adjusted model		Unadjusted model		Adjusted model		Unadjusted model		Adjusted model	
		SM = 0.794				SM = 0.875				SM = 0.680				SM = 0.675			
Ethnicity	Māori	-0.013	(0.019)	-0.026	(0.021)	-0.017	(0.016)	0.008	(0.018)	-0.037^a^	(0.020)	-0.009	(0.022)	-0.087^c^	(0.019)	-0.064^c^	(0.022)
	Pacific peoples	0.116^c^	(0.021)	0.096^c^	(0.026)	-0.086^c^	(0.014)	-0.065^c^	(0.019)	0.062^c^	(0.021)	0.049^b^	(0.025)	-0.003	(0.020)	-0.006	(0.023)
	Asian	0.088^c^	(0.019)	0.098^c^	(0.024)	-0.068^c^	(0.013)	-0.067^c^	(0.018)	-0.003	(0.019)	-0.011	(0.024)	-0.039^b^	(0.019)	-0.056^b^	(0.024)
	Other ethnicity	0.008	(0.032)	0.016	(0.033)	0.001	(0.028)	0.009	(0.029)	-0.014	(0.035)	-0.013	(0.036)	0.068^a^	(0.037)	0.054	(0.038)
Mother	Current age (years)			-0.004^c^	(0.001)			-0.001	(0.001)			0.002	(0.001)			0.002	(0.001)
	Current weight (kg)			0.001^b^	(0.000)			-0.001^c^	(0.000)								
	Disability status			0.049^a^	(0.026)			-0.028	(0.019)			-0.011	(0.032)			0.029	(0.032)
	Smokes regularly			-0.043^a^	(0.023)			-0.038^b^	(0.018)			-0.028	(0.021)			-0.017	(0.021)
Child	Girl											0.001	(0.013)			0.000	(0.013)
	Birthweight (grams)											0.000	(0.000)			-0.00001	(0.000)
	First child			0.020	(0.015)			-0.017	(0.012)			-0.001	(0.017)			-0.009	(0.016)
	Child health/developmental concern											0.004	(0.021)			-0.038	(0.024)
Socio-economic	Mother’s employment			-0.003	(0.014)			0.007	(0.011)			-0.030^b^	(0.014)			-0.015	(0.014)
	Mother’s post-graduate qualification			0.011	(0.016)			0.007	(0.014)			-0.024	(0.018)			0.025	(0.018)
	Household income < NZ$ 50 K (*Omitted* > *100 K*)			-0.044^b^	(0.02)			-0.013	(0.016)			-0.038^b^	(0.020)			-0.036^a^	(0.020)
	Household income > = NZ$ 50 K & < = 100 K			-0.02	(0.014)			-0.003	(0.012)			-0.040^c^	(0.016)			-0.039^b^	(0.016)
Household	Partner			-0.047	(0.037)			0.029	(0.027)			0.005	(0.027)			-0.008	(0.024)
	NZ born mother			0.012	(0.016)			0.001	(0.013)			-0.008	(0.017)			-0.018	(0.017)
	Household size (number)			0.002	(0.005)			0.004	(0.004)								
	Number of people > = 18 years											0.001	(0.007)				
	Number of people < 18 years											0.001	(0.008)				
	Number of siblings															0.000	(0.006)
Mobility	Numbers of residential moves			0.006	(0.004)			0.000	(0.003)			-0.012^c^	(0.004)			0.006	(0.010)
	Rural location			-0.055^c^	(0.021)			0.02	(0.020)			-0.064^c^	(0.024)			-0.104^c^	(0.023)
	Local healthcare											-0.029	(0.015)				
	Mother self-driving			0.010	(0.017)			0.022^a^	(0.013)			0.02	(0.018)			0.040^a^	(0.022)
	Internet access																
Social	Discriminated against			0.014	(0.015)			-0.021^a^	(0.012)			-0.064^c^	(0.015)			-0.056^c^	(0.022)
	Discouraged to immunise			-0.006	(0.018)			-0.017	(0.014)			-0.056^c^	(0.019)			-0.034^a^	(0.020)
	Encouraged to immunise			0.037^c^	(0.013)			-0.001	(0.011)			0.035^c^	(0.014)			0.026^a^	(0.014)
	Childcare services											0.002	(0.020)			-0.013	(0.015)
	Observations	4527		4527		4527		4527		5341		5341		5332		5332	

The above table presents marginal effects from Probit models. The robust standard errors are reported within parentheses

*SM* Sample mean

^c^, ^b^, ^a^denote the coefficients are significantly different from zero at the 1%, 5%, and 10% level respectively. The ethnicity information is derived from self-prioritised ethnicity

Additional factors associated with lower primary care uptake and satisfaction were maternal smoking, low household income, rural location, feeling discriminated against, and being discouraged to immunise. Factors associated with higher uptake and satisfaction were higher mobility (self-driving) and being encouraged to immunise, while negative social feedback was associated with lower access and satisfaction. 

#### Explaining the gap between Māori and NZ European

In Table 4, we decompose the observed differences in outcomes between NZ European and Māori (top half of table) and between NZ European and Pacific (bottom half of table) across all four survey waves. As indicated in Table 1, all independent variables are classified into six categories – mother, child, socio-economic, household, mobility, and other social aspects. Table 4 shows how much of the total ethnic difference in healthcare outcomes were explained by the covariates included along with the respective share of each category in the explained difference.

**Table 4:** Decomposition of the ethnic differences, comparisons for nz european-māori and nz european-pacific peoples

	Immunisations				Primary care			
	Intend to fully immunise	All first-year immunisations on time	Received 15-month immunisations	Received age 4 immunisations	First choice LMC	Seen doctor/ GP while pregnant	Satisfied with usual GP practice (9-months)	Satisfied with usual GP practice (age 2)
***Māori*—Vector of covariates**	(1)	(2)	(3)	(4)	(5)	(6)	(7)	(8)
Mother	-0.040	0.013	-0.003	-0.003	0.014	-0.007	0.008	0.005
Child	-0.017	0.007	0.003	0.008	-0.001	0.004	0.002	-0.003
Socio-economic	0.016	0.029	0.005	0.002	0.008	0.009	0.007	0.022
Household	-0.004	0.068	-0.005	0.017	-0.008	-0.001	0.004	0.009
Mobility	0.001	-0.001	-0.001	0.004	0.002	-0.003	-0.001	-0.006
Social	-0.008	-0.004	-0.002	-0.008	0.008	-0.010	0.010	0.003
**Explained**	-0.051	0.115	-0.004	0.020	0.023	-0.007	0.029	0.03
**Total difference (NZ European—Māori)**	-0.059	0.169	-0.003	0.039	0.015	0.014	0.038	0.09
**Percent explained**	86.40%	68.00%	133.30%	51.30%	153.30%	-50.00%	76.30%	33.30%
N_European_	2682	3165	2976	3111	2682	2682	3140	3113
N_Māori_	513	672	644	563	513	513	657	672
***Pacific peoples* Vector of covariates**								
Mother	-0.034	0.009	0.000	0.002	0.005	-0.016	0.011	0.012
Child	-0.024	0.016	0.005	0.015	-0.004	0.003	0.005	-0.002
Socio-economic	0.032	0.035	0.008	0.003	0.012	0.023	0.000	0.009
Household	-0.029	0.078	-0.001	0.026	-0.006	-0.002	-0.013	-0.011
Mobility	-0.002	-0.012	-0.002	-0.005	0.002	0.000	-0.017	-0.009
Social	-0.035	-0.019	-0.006	-0.013	0.003	-0.015	-0.009	-0.003
**Explained**	-0.093	0.107	0.004	0.029	0.003	-0.006	-0.023	-0.003
**Total difference (NZ European – Pacific)**	-0.190	0.055	-0.020	-0.057	0.096	-0.104	-0.059	0.004
**Percent explained**	48.90%	194.50%	-20%	-50.90%	3.10%	5.80%	39.00%	-75.00%
N_European_	2683	3165	2976	3103	2683	2683	3140	3113
N_Pacific peoples_	514	630	632	422	514	514	630	647

The above table reports estimates of each of the vectors’ contribution in explaining the observed differences in health outcomes between NZ Europeans and Māori and between NZ Europeans and Pacific peoples. The non-linear decomposition employs a pooled probit model that considers only observations from the two ethnicities compared in the analysis

For immunisation coverage, the total ethnic difference in antenatal intention to immunise is only -0.059, with the negative sign indicating Māori having higher intention to immunise than NZ European. Approximately 86% of the gap can be explained by the covariates incorporated in our analysis (-0.051 out of -0.059). A substantial proportion of the ‘explained’ difference is driven by maternal characteristics (-0.040, (82%)). For timely immunisations observed at 9-month survey, Māori children had lower coverage than NZ Europeans. Almost 68% (0.115 out of 0.169) of the total ethnic gap could be explained by the independent variables. Household characteristics accounted for 40% of the gap. At 2-years, the ethnic gap in immunisations between Māori and NZ European is small (-0.003), and more than fully explained by the covariates included. At 4-years, we find that 51% of the total immunisation gap is explained (0.020 out of a total of 0.039) by the independent variables and is primarily driven by household characteristics (explains 44%).

The gap in first choice LMC is over-explained by the independent variables and mostly driven by maternal characteristics. In terms of the primary care-related outcomes, results vary depending on outcome of interest and time point. For instance, 76% of the ethnic gap in satisfaction with GP can be explained when the child is 9-months old, but this falls to 33% when the child is 2-years old. At 9-months, social factors are the largest contributor towards the explained gap in satisfaction with GP satisfaction. These factors include perceived discrimination, as well as external sources of both encouragement and discouragement towards immunisations.

#### Explaining the gap between Pacific peoples and NZ European

The negative total difference in antenatal intention to immunise between Pacific Peoples and NZ European indicates that Pacific mothers have higher intention to immunise their children (Table 4). The factors included in the model explain 49% of the antenatal gap. Pacific children are also more likely to be fully immunised by 2-years and by 4-years. However, the respective differences are under-explained in the decomposition model indicating that the unexplained (unobserved) difference substantially exceeds the explained difference. The difference in timely immunisation at the 9-month survey is more than fully explained by the covariates included in the decomposition analysis (195%). This indicates that if Pacific households had the same observable characteristics/household resources of the population represented by the pooled sample of both the ethnic groups, their immunisation timeliness at the 9-month stage would have exceeded that of NZ Europeans.

For primary care-related outcomes, Pacific mothers are less likely to have their first choice for LMC compared to NZ Europeans. However, the individual and household level variables included in the model explain only 3% of this ethnic difference. Pacific mothers are more likely to be satisfied with their GP at 2-years, and 39% of the ethnic gap is explained by the model. The total ethnic gap for the same outcome at 4-years remains mostly unexplained.

## Discussion

Overall, in crude analyses, there were ethnic differences in the uptake of immunisation and primary healthcare. NZ European mothers had higher rates of child healthcare utilisation for some indicators relative to other ethnic groups, but there was not a consistent pattern. After adjusting for individual, socio-economic, and social covariates, some gaps remained. Pacific and Asian children had higher immunisation uptake at all time-points compared to NZ European children, while Māori children had lower timeliness of first-year immunisations. For primary care, Pacific and Asian mothers were more likely to have seen a GP during pregnancy, but less likely to have their first choice LMC compared to NZ European mothers. Pacific mothers were more likely to be satisfied, and Māori and Asian mothers were less likely to be satisfied with their GP at 2-years.

Māori mothers had higher intention to immunise than NZ European mothers, but lower timeliness of first-year immunisations and GP satisfaction at 2-years. This suggests that structural and institutional factors may be important barriers to healthcare utilisation for Māori. This aligns with the findings of the WAI 2575 report from the Waitangi Tribunal, which describes the legacies of colonisation on health inequities [33]. Using decomposition analysis, we found that two-thirds of the ethnic gap in the immunisation timeliness between NZ European and Māori could be explained by the independent variables included in the analysis, with a substantial portion of the difference being driven by household characteristics, including whether the mother was parenting alone, and number of siblings in the household. These may be indicators of managing the multiple demands of the family and household responsibilities. Less of the difference in GP satisfaction was explained by the model. Socio-economic factors like employment, education, and income contributed the most to the ethnic gap in GP satisfaction, and may indicate a lack of choice and inability to change their GP by Māori parents. While previous experience of ethnic discrimination was an important determinant of less primary care choice and satisfaction in multivariable models, it did not contribute substantially to ethnic gaps in decomposition analysis.

The main negative disparities for Pacific compared to NZ European were timeliness of first-year immunisations and first choice LMC. The independent variables included in the analysis over-explained the ethnic gap for immunisation timeliness. As for Māori, this was mainly due to household characteristics. The explained component was very low for first choice LMC. Social and/or structural causes may underly late booking, which in turn leads to limited choice [34]. Further research to explore other factors that may be driving ethnic differences beyond the covariates used in these analyses would be beneficial and is underway [35].

The finding that Asian and Pacific children had the highest immunisation uptake at all time-points, along with children of non-NZ born mothers, is consistent with other research. Qualitative findings from interviews with immigrant mothers in the Netherlands perceived childhood vaccination to be self-evident and important [36]. Fear of vaccine-targeted diseases was a key motivating factor for immigrant parents adopting vaccination [37, 38], along with higher perceptions of vaccine safety [6, 39]. Qualitative research in NZ could learn from Asian and Pacific mothers to improve vaccination uptake in other ethnic groups.

However, there was a large raw gap in timely immunisation for Māori and Pacific children. Household and socioeconomic factors combined contributed the largest part to the explained differences in immunisation timeliness. Mothers being employed, parenting alone, and having other young children to look after could be related to time-constraints. Families with limited income may not be able to afford to take long parental leave, and other analyses of GUiNZ data have found that mothers who didn’t take any leave at all were more likely to be younger, parent alone, Māori, have low paid employment, and be from low-income families [40]. This pattern is consistent with research in other countries [7, 20, 22, 23].

Going to a childcare service is associated with a greater likelihood of vaccinating on time (5 percentage points), and higher complete immunisation at 4-years (7 percentage points). Childhood services may require parents to follow preventive guidelines, and provide proof of vaccination status in order to protect enrolled children’s health. There are likely to be inter-related ethnic and socio-economic differences in use of childcare services. Migrant families may use childcare services more, as they have fewer extended family options for childcare, combined with economic pressure and visa requirements to continue working.

A higher number of residential moves is also associated with lower income, and is higher among Māori and Pacific families [19]. In this study residential mobility was associated with less timely first-year immunisations, lower complete immunisation at 4-years, and lower GP satisfaction at 2-years. This may be due to inability to access new primary care providers (GP) easily, to move from one provider to another, or to negotiate travel to their existing GP. Registering with a new GP service can be complex and costly. Given the increased mobility of lower income Māori and Pacific families, strategies to improve timely access to free and flexible /unregistered primary care are required.

Social influences, such as discouragement or encouragement regarding child immunisation were important determinants of immunisation uptake, as well as GP use and satisfaction. This suggests that there may be social influences on atittudes towards healthcare more generally as well as forspecific services like immunisation. Our results align with previous GUiNZ research showing that compared to mothers who didn’t receive any encouraging or discouraging information, receiving discouragement is negatively associated with the likelihood that child was immunised on time [41]. Social norms also have a key role in influencing parental decision-making around vaccination, and could contribute to ethnic differences [37]. In our study, ethnic-specific models showed that discouragement had a negative effect on immunisation for NZ Europeans and Māori, but not Pacific peoples and Asians. A systematic review of determinants of measles vaccination uptake in European countries, found that negative perceptions and attitudes towards vaccination were important, alongside household and socio-economic factors [21]. Perceived ethnically motivated discrimination by a health professional was associated with reduced primary healthcare satisfaction in our study, and elsewhere [29]. Indeed, social factors explained a large part of the ethnic gap in first choice LMC.

This analysis informs debate on ethnic disparities in use of child healthcare services in NZ, but there are a number of limitations. First, the characteristics of the cohort sample may not be representative of the whole of NZ. For example, on average, the academic qualification level of the mothers in our sample (aged 18 to 41) is higher than the national average [42]. Future analyses should focus on a wider population-based sample of mothers. Second, given the saturated nature of our multivariable regression models, it is plausible that some of our covariates are highly correlated, thereby potentially affecting both the estimation and precision of our regression coefficients. However, after performing standard additional diagnostics on this front, we found no statistical evidence in support of the presence of multicollinearity. Third, the variables related to antenatal GP use may conflate both underlying differences in health status as well as differences in unmet need for healthcare. Future analyses differentiating these two components would provide further insight into ethnic differences in the uptake of healthcare. The final limitation is that the results do not represent causal relationships, but our findings do provide ideas for future research to inform public health and policy interventions.

Future research could use this birth cohort to further examine causal mechanisms and explore persistence of healthcare utilisation behaviours over time – particularly for immunisation, where there are four time points of data available. Other possible determinants of health service uptake such as service provider characteristics could also be explored [43]. We have conducted qualitative research to better understand the reasons for underutilisation of childhood healthcare services, to explore other factors that might explain ethnic gaps, and give insights to inform policy approaches to address these gaps. Research among groups with higher uptake, including with Pacific and Asian families around immunisation, could be harnessed to improve uptake in other groups. Although Asian children had some of the best healthcare utilisation indicators, this group includes considerable diversity, and grouping them together may mask heterogeneity that is worth exploring.

## Conclusions

Influences on childhood healthcare utilisation go beyond health service provision, and require broader consideration of the social determinants of disparities, confronting structural racism, and decolonising health systems. There are some important differences in determinants of healthcare utilisation by ethnic group, and interventions may need to be tailored to these to address underlying ethnic gaps. Potential candidates for policy levers identified by this study include addressing ethnic-specific social influences in immunisation uptake and satisfaction with GP. Specific policies to supporting caregivers on low incomes, who are parenting alone, caregivers with several young children, especially those not attending childcare services, families in rural areas, and those who move house frequently would help to reduce gaps. In addition, moves between GP practices should be facilitated, and payments for non-enrolled clients removed. Addressing discriminatory practices and increasing cultural safety in primary healthcare services would increase satisfaction, particularly for Māori families.

## Supplementary Information

**Additional file 1.**

## Data Availability

The data that support the findings of this study are not publicly available, but can be obtained from Growing Up in New Zealand. The data used in this work is governed by a Data Access Protocol to ensure that only qualified researchers access the information and to safeguard the privacy of study participants and their families. This data may be accessed in four ways, and the method used in this study was anonymised data sets and supporting documents including videos, data dictionaries and user guides. Anyone who wants to use *Growing Up in New Zealand* data needs to make a formal Data Access Application. The process is outlined here: https://www.growingup.co.nz/access-growing-data. The Growing Up in New Zealand Kaitiaki principles that provide a framework for ensuring that Māori rights and aspirations for research and policy development are upheld can be accessed here: https://www.tandfonline.com/doi/abs/10.1080/03036758.2022.2066142.

## Abbreviations

DCW: Data collection wave
GP: General Practitioner
GUiNZ: Growing Up in New Zealand birth cohort study
LMC: Lead maternity caregiver
MELAA: Middle Eastern, Latin American, and African
NIR: National Immunisation Register
NZ: New Zealand
