# Supplementary material for: Determinants of ethnic differences in the uptake of child healthcare services in New Zealand: a decomposition analysis
Source: Int J Equity Health. 2023 Jan 16;22:13. doi: 10.1186/s12939-022-01812-3 (PMC9841674; doi:10.1186/s12939-022-01812-3)
Supplement: Supplementary file 1 — Additional file 1. [file 12939_2022_1812_MOESM1_ESM.docx]

# **Supplementary material**

## Statistical analysis to identify ethnic differences in healthcare access

First we looked for associations between our outcome measures of interest, and a range of factors including mother and child characteristics, socio-economic status, household characteristics, mobility, and other social aspects (such as discrimination). We used multivariable probit regression to explore the relationships between the outcomes and covariates.

As the outcome variables were binary, we used non-linear models. For each survey wave $t$ (DCW0, DCW1, DCW2, and DCW4) which captures the life-course from the antenatal period through to when the child is age 4, we estimated the following reduced form model:

$y_{it}=\mathbf{1}(\alpha_{t}+ \beta_{t}\mathrm{Ethnicit}y_{i}+Z_{it}^{'}\gamma\mathbf{+} u_{it}>0)$ (1)

Where $y_{it}$ is the binary outcome of interest related to mother $i$ (note that there are only single child-mother pairs) for survey wave $t$. As illustrated in Table 1, outcomes include six variables capturing immunisation intention and uptake, and four variables encompassing both use of healthcare practitioners and satisfaction with those practitioners. Further, $\mathrm{Ethnicit}y_{i}$ represents mother’s self-prioritised ethnicity (note that the respective coefficient $\beta_{t}$ is estimated separately for each survey wave. $Z_{it}^{'}$ is a vector of all other individual-level characteristics of mother and child (as discussed in the previous section). Finally, $u_{it}$ is the error term that captures unobserved endowments and $u_{it}\sim N\left( 0,\sigma_{u}^{2} \right)$. Since $y_{it}$ is binary, a normalization is required and we chose $\sigma_{u}^{2}=1$. The outcome probabilities are:

$P_{it}\left( y_{it}=1 \right)=\Phi\left[ (\alpha_{t}+ \beta_{t}\mathrm{Ethnicit}y_{i}+Z_{it}^{'}\gamma)\left( 2y_{it}-1 \right) \right]$ (2)

With $\Phi$ being the cumulative standard normal distribution function.

## Statistical analysis to explain ethnic differences in healthcare access

Next we quantified the contribution of each of these sets of factors to understand what proportion of the ethnic gap they explained. We built on the descriptive evidence obtained from the multivariable analysis, by evaluating the contribution of the different covariates in explaining observed ethnic differences in indicators of health service uptake. Given the dichotomous nature of our dependent variables, we use the Fairlie decomposition method (see Fairlie 2005) which extends the standard Blinder-Oaxaca decomposition method for application in non-linear models. We employ a pooled probit specification by using the user-written ‘Fairlie’ package in Stata16 developed by Jann (2006). The pooled specification allows us to estimate the coefficients by pooling variables from both the groups compared.

For our analysis, we compare Māori and Pacific peoples respectively, to NZ Europeans. In general, the Fairlie decomposition model can be represented by:

$\bar{Y}^{E}- \bar{Y}^{NE}= \left[ \sum_{i=1}^{N^{E}} \frac{F\left( X_{i}^{E}.\hat{\beta}^{E} \right)}{N^{E}}- \sum_{i=1}^{N^{NE}} \frac{F\left( X_{i}^{NE}.\hat{\beta}^{E} \right)}{N^{NE}} \right]+ \left[ \sum_{i=1}^{N^{NE}} \frac{F\left( X_{i}^{NE}.\hat{\beta}^{E} \right)}{N^{NE}}- \sum_{i=1}^{N^{NE}} \frac{F\left( X_{i}^{NE}.\hat{\beta}^{NE} \right)}{N^{NE}} \right]$(3)

Where,$\bar{Y}^{E}- \bar{Y}^{NE}$ represents the actual difference in the outcome between NZ European (indexed by superscript *E*) and each of the non-NZ European groups (indexed by superscript *NE*). Indexed by group-specific superscripts, $X$,$\hat{\beta},$ and $N$ represent the vector of the average values of covariates (used in aim 1 analysis), corresponding estimated regression coefficients and sample size, respectively.

The first summand on the right-hand side of equation represents the part of the ethnic gap that is ‘explained’ by ethnic group differences in distributions of the vector of covariates. The second term isn’t straightforward to interpret and represents the ‘unexplained’ part - it incorporates the portion of the ethnic differences that is driven by across-group differences in unobserved influences.

Table A. 1 - Ethnic-specific analysis of all first-year immunisations on time (9-month survey)

|  | Variables | NZ European | | Māori | | Pacific peoples | | Asian | |
| --- | --- | --- | --- | --- | --- | --- | --- | --- | --- |
|  |  | SM = 0.715 | | SM =0.546 | | SM = 0.660 | | SM = 0.907 | |
| Mother & Child | Girl | 0.008 | (0.015) | 0.000 | (0.037) | 0.044 | (0.036) | 0.00 | (0.026) |
|  | Birthweight (in grams) | -0.00003^**^ | (0.00001) | -0.00002 | (0.00003) | 0.00005^*^ | (0.00003) | -0.00001 | (0.00002) |
|  | First child | 0.075^***^ | (0.021) | 0.096^**^ | (0.047) | 0.200^***^ | (0.049) | 0.022 | (0.036) |
|  | Child health/developmental problem | -0.050^**^ | (0.023) | 0.052 | (0.067) | -0.113^*^ | (0.068) | -0.073 | (0.056) |
|  | Mother’s age | 0.002 | (0.002) | 0.006^*^ | (0.004) | 0.003 | (0.004) | -0.001 | (0.003) |
|  | Mother’s disability | 0.031 | (0.039) | -0.022 | (0.078) | 0.118 | (0.142) | 0.121 | (0.079) |
|  | Regular smoker | -0.018 | (0.017) | -0.048 | (0.040) | 0.000 | (0.045) | -0.094 | (0.100) |
| Socio-economic | Employment | 0.022 | (0.020) | -0.042 | (0.045) | 0.075^*^ | (0.043) | -0.018 | (0.028) |
|  | Post-graduation | 0.021 | (0.020) | -0.121 | (0.086) | -0.077 | (0.099) | -0.027 | (0.034) |
|  | Household income <NZ$ 50K (*Omitted >100K*) | -0.135^***^ | (0.025) | -0.063 | (0.060) | 0.066 | (0.067) | -0.039 | (0.043) |
|  | Household income >=NZ$ 50K & <=100K | -0.087^***^ | (0.018) | -0.035 | (0.057) | 0.071 | (0.065) | -0.046 | (0.039) |
| Household | Partner | -0.004 | (0.038) | 0.036 | (0.055) | -0.004 | (0.053) | 0.081 | (0.080) |
|  | NZ born | -0.002 | (0.019) | -0.053 | (0.123) | -0.028 | (0.041) | -0.044 | (0.062) |
|  | Number of people >=18 years | -0.011 | (0.013) | 0.007 | (0.016) | -0.002 | (0.014) | 0.001 | (0.011) |
|  | Number of people <18 years | -0.081^***^ | (0.011) | -0.105^***^ | (0.015) | -0.042^***^ | (0.014) | -0.036 | (0.021) |
| Mobility | Numbers of residential moves | -0.011^**^ | (0.005) | -0.017 | (0.011) | -0.015 | (0.013) | -0.014 | (0.009) |
|  | Rural location | -0.012 | (0.024) | 0.036 | (0.070) | -0.010 | (0.197) | - | - |
|  | Local healthcare | 0.041^**^ | (0.017) | 0.074^*^ | (0.043) | -0.017 | (0.041) | 0.013 | (0.030) |
|  | Drove car | -0.011 | (0.027) | 0.017 | (0.052) | -0.038 | (0.043) | 0.002 | (0.029) |
| Other social  aspects | Discriminated against | -0.012 | (0.020) | -0.057 | (0.037) | -0.052 | (0.044) | 0.060^*^ | (0.031) |
|  | Discouraged to immunise | -0.170^***^ | (0.021) | -0.159^***^ | (0.055) | 0.087 | (0.078) | -0.087 | (0.060) |
|  | Encouraged to immunise | 0.021 | (0.018) | 0.020 | (0.039) | 0.001 | (0.037) | 0.003 | (0.026) |
|  | Childcare services | 0.084^***^ | (0.024) | -0.019 | (0.053) | -0.013 | (0.063) | 0.016 | (0.045) |
|  | Observations | 3165 | | 672 | | 630 | | 732 | |

Notes: The above table presents marginal effects from probit models. The robust standard errors are reported within parentheses. SM = Sample mean. ^***^, ^**^, ^*^ denote the coefficients are significantly different from zero at the 1%, 5%, and 10% level respectively. The ethnicity information is derived from self-prioritised ethnicity.

Table A. 2 - Age-specific analysis of all first-year immunisations on time (9-month survey)

|  | Variables | 18 ≤ Age < 30 | | Age 30 & above | |
| --- | --- | --- | --- | --- | --- |
|  |  | SM = 0.687 | | SM = 0.726 | |
| Ethnicity | Māori | -0.056^**^ | (0.025) | -0.061^**^ | (0.030) |
|  | Pacific Peoples | 0.041 | (0.031) | 0.031 | (0.034) |
|  | Asian | 0.078^**^ | (0.037) | 0.091^***^ | (0.034) |
|  | Other Ethnicity | 0.030 | (0.056) | 0.086^**^ | (0.043) |
| Mother & Child | Girl | 0.012 | (0.018) | 0.014 | (0.016) |
|  | Birthweight | -0.00002 | (0.00002) | -0.00001 | (0.00001) |
|  | First child | 0.100^***^ | (0.022) | 0.072^***^ | (0.022) |
|  | Child health/developmental problem | -0.013 | (0.030) | -0.072^***^ | (0.025) |
|  | Mother’s age | 0.001 | (0.003) | -0.002 | (0.003) |
|  | Mother’s disability | -0.016 | (0.045) | 0.059 | (0.039) |
|  | Regular smoker | -0.025 | (0.024) | -0.021 | (0.031) |
| Socio-economic | Employment | -0.010 | (0.021) | -0.024 | (0.017) |
|  | Post-graduation | -0.032 | (0.032) | 0.008 | (0.019) |
|  | Household income <NZ$ 50K (*Omitted >100K*) | -0.118^***^ | (0.032) | -0.073^***^ | (0.025) |
|  | Household income >=NZ$ 50K & <=100K | -0.074^***^ | (0.029) | -0.067^***^ | (0.018) |
| Household | Partner | 0.002 | (0.031) | 0.037 | (0.040) |
|  | NZ born | -0.057^**^ | (0.026) | -0.015 | (0.020) |
|  | Number of people >=18 years | 0.003 | (0.008) | -0.011 | (0.011) |
|  | Number of people <18 years | -0.069^***^ | (0.010) | -0.070^***^ | (0.009) |
| Mobility | Numbers of residential moves | -0.018^***^ | (0.006) | -0.007 | (0.005) |
|  | Rural location | 0.009 | (0.032) | -0.045 | (0.029) |
|  | Local healthcare | 0.032 | (0.020) | 0.039^**^ | (0.018) |
|  | Drove car | -0.025 | (0.024) | 0.018 | (0.026) |
| Other social  aspects | Discriminated against | -0.001 | (0.020) | -0.018 | (0.020) |
|  | Discouraged to immunise | -0.135^***^ | (0.026) | -0.158^***^ | (0.023) |
|  | Encouraged to immunise | 0.011 | (0.019) | 0.023 | (0.018) |
|  | Childcare services | 0.045^*^ | (0.028) | 0.050^**^ | (0.024) |
|  | Observations | 2453 | | 2931 | |

Notes: SM = Sample mean. ^***^, ^**^, ^*^ denote the coefficients are significantly different from zero at the 1%, 5%, and 10% level respectively. The ethnicity information is derived from self-prioritised ethnicity.

Table A.3 - Regression analysis comparing administrative record and self-reported measure of 6week/3months/5months vaccination

|  |  |  | All first-year immunisations  (NIR-validated) | |  | All first-year immunisations  (Self-reported) | |
| --- | --- | --- | --- | --- | --- | --- | --- |
|  |  | n (proportion) | Marginal effects (SE) | | n (proportion) | Marginal effects (SE) | |
|  | Total | 6668 (0.88) |  |  | 6846 (0.84) |  |  |
| Ethnicity | NZ European *(Omitted)* | 3550 (0.88) | - | - | 3642 (0.86) | - | - |
|  | Māori | 924 (0.81) | -0.007 | 0.013 | 947 (0.74) | -0.019 | 0.013 |
|  | Pacific peoples | 970 (0.89) | 0.067^***^ | 0.017 | 997 (0.80) | 0.063^***^ | 0.017 |
|  | Asian | 974 (0.94) | 0.039^**^ | 0.019 | 1002 (0.88) | 0.042^**^ | 0.019 |
|  | Other ethnicity |  | 0.003 | 0.024 |  | 0.002 | 0.023 |
| Mother & Child | Girl |  | 0.015^*^ | 0.008 |  | -0.005 | 0.008 |
|  | Birthweight (grams) |  | -0.00003^***^ | 0.000007 |  | -0.00002^***^ | 0.000007 |
|  | First child |  | 0.025^**^ | 0.011 |  | 0.037^***^ | 0.011 |
|  | Child health/developmental problem |  | 0.016 | 0.014 |  | -0.014 | 0.013 |
|  | Mother’s age |  | 0.001 | 0.001 |  | 0.001 | 0.001 |
|  | Mother’s disability |  | 0.039^*^ | 0.021 |  | 0.028 | 0.021 |
|  | Regular smoker |  | 0.005 | 0.013 |  | 0.006 | 0.013 |
| Socio-economic | Employment |  | 0.011 | 0.009 |  | 0.012 | 0.009 |
|  | Post-graduation |  | 0.013 | 0.012 |  | 0.015 | 0.012 |
|  | Household income <NZ$ 50K (*Omitted >100K*) |  | -0.050^***^ | 0.013 |  | -0.043^***^ | 0.013 |
|  | Household income >=NZ$ 50K & <=100K |  | -0.031^***^ | 0.011 |  | -0.023^***^ | 0.011 |
| Household | Partner |  | 0.009 | 0.017 |  | 0.018 | 0.017 |
|  | NZ born |  | -0.027^**^ | 0.012 |  | -0.020^*^ | 0.012 |
|  | Number of people >=18 years |  | 0.000 | 0.005 |  | 0.002 | 0.005 |
|  | Number of people <18 years |  | -0.035^***^ | 0.004 |  | -0.029^***^ | 0.004 |
| Mobility | Number of residential moves |  | -0.007^***^ | 0.003 |  | -0.004 | 0.003 |
|  | Rural location |  | -0.022 | 0.014 |  | -0.033^**^ | 0.014 |
|  | Local healthcare |  | 0.030^***^ | 0.009 |  | 0.007 | 0.009 |
|  | Self-driving |  | -0.004 | 0.013 |  | 0.002 | 0.012 |
| Other social aspects | Discriminated against |  | -0.004 | 0.010 |  | -0.005 | 0.010 |
|  | Discouraged to immunise |  | -0.096^***^ | 0.011 |  | -0.099^***^ | 0.011 |
|  | Encouraged to immunise |  | 0.013 | 0.009 |  | 0.016^*^ | 0.009 |
|  | Childcare services |  | 0.013 | 0.013 |  | -0.012 | 0.012 |
|  | Observations |  | 5384 | |  | 5511 | |
